# Impact of COVID-19 Lockdown on Sun Exposure of UK Office Workers

**DOI:** 10.3390/ijerph18084362

**Published:** 2021-04-20

**Authors:** Katarzyna Anna Baczynska, Rebecca J. Rendell, Marina Khazova

**Affiliations:** Centre for Radiation, Chemical and Environmental Hazards, Public Health England, Chilton, Oxfordshire OX11 0RQ, UK; Becky.Rendell@phe.gov.uk (R.J.R.); Marina.Khazova@phe.gov.uk (M.K.)

**Keywords:** sun exposure, UV exposure, office workers, UV radiation

## Abstract

The impact of lockdown due to the COVID-19 pandemic in April–June 2020 on UV exposure of office workers was assessed using an online survey on time spent outdoors and environmental data for different locations in the UK. Without the need for commuting and with the flexibility of homeworking, weekday time spent outdoors was higher in the 2020 lockdown than in the same period in 2017. The weekday erythema effective radiant exposure was higher in 2020 due to an additional 45 min outdoors in the late afternoon that was not observed in 2017 and high UV levels due to extremely sunny weather in spring. The lockdown did not impact the frequency of time spent outdoors around midday, which was still governed by work commitments, and at the weekends, no difference between 2020 and 2017 was observed. In 2020, responders felt that time outdoors was very important for their health and well-being.

## 1. Introduction

There is a consensus in the international community that overexposure to solar radiation can cause considerable damage to the skin and the eye, increase the risk of skin cancers, and suppress adaptive immunity [1,2,3,4,5,6]. Established and emerging health benefits from sun exposure include cutaneous vitamin D synthesis and its importance for health [7], as well as the role of UV-A and visible sunlight in blood pressure modulation, and melatonin and serotonin regulation for mood and cognition [8,9]. Sun exposure depends on an individual’s behaviour [10,11] and ambient levels that change with latitude and altitude, the time of year and day, and meteorological conditions [12]. For the working population, weekday sun exposure is governed by work patterns, family commitments, and lifestyle. The previous observational study on time spent outdoors by UK office workers in summer 2017 [13] showed that the majority of survey responders received negligible weekday erythema effective radiant exposure that was restricted by commuting and office hours and was limited to early mornings and evenings when UV levels are lowest.

On 23 March 2020, the UK government introduced wide-ranging lockdown measures to mitigate the SARS-CoV-2 virus spread including the closure of non-essential shops, a ban on public gatherings, and severe restrictions to people’s movement which remained in place until 11 May 2020 [14]. The government effort had an immediate effect on office workers with advice for homeworking when possible. The UK and international travel were also restricted. From mid-May, some restrictions started to be eased and that continued throughout the summer, but the prevalence of homeworking among office workers remained high, and it is expected to extend beyond the pandemic. Sun exposure during this period could also have been affected by exceptionally sunny weather in the UK in spring 2020: April and May had the highest sunshine hours on record across the country [15].

A number of lifestyle surveys [16,17] were used to measure the impact of lockdown on physical activity and well-being of the British public. However, these surveys mostly collected information on the duration of physical exercises but not the time of the day or location, indoors or outdoors. The impact of the lockdown on time spent in green and natural spaces was also assessed [18], and this information, combined with detailed environmental data, can provide valuable input to an analysis of sun exposure during the lockdown.

This paper assesses the impact of the UK lockdown in April–June 2020 on UV exposure of the UK office workers using a survey on time spent outdoors and environmental data for four locations in the UK.

## 2. Materials and Methods

Office and laboratory workers at Public Health England (PHE) were asked to complete a questionnaire about the time spent outdoors. The research study approval was granted by the PHE Research Ethics and Governance Group. PHE is the UK government organisation employing 5584 staff (on 20 March 2020) that could be considered as office or laboratory staff and included scientists, public health professionals, and administrative personnel. (of whom 68% were female and 32% were male). From the beginning of the lockdown, up to 77.4% of PHE staff worked from home, and up to 9.8% mixed homeworking with office or lab-based work. The prevalence of homeworking did not substantially change over the May–August 2020 period when the lockdown restriction started to ease.

The invitation to take part in the survey was included in the PHE internal Weekly Newsletter, together with other information, and sent to all staff on 27 May 2020; it was followed by one reminder three weeks later, one week before the survey closure. A total of 12 questions were asked as follows:

- On average, on how many days per week do you spend time outdoors in:
  ∘ the morning before 11:00;
  ∘ midday, 11:00–15:00;
  ∘ in the afternoon, 15:00–18:00;
  ∘ in the evening after 18:00.

i.e., never, occasionally, once, twice, three or four times a week?

- On these days, how much time on average do you spend outdoors in the morning/afternoon/evening, i.e., none, up to 30 min, 30–60 min, 60–90 min, or more than 90 min?
- On these days, how much time on average do you spend outdoors in the midday, i.e., up to 15 min, 15–30 min, 30–45 min, 45–60 min, or more than 60 min?
- What time do you go outdoors in the morning, i.e., before 9:00, 9:00–10:00, and/or 10:00–11:00?
- At the weekend, how much time in total over the two days do you spend outdoors, i.e., less than 2 h, 2–4 h, 4–6 h, 6–8 h, 8–10 h, or more than 10 h?
- What is your gender, e.g., male, female, gender variant/non-conforming, or I prefer not to say?
- What is your age group, i.e., under 25, 25–45, 46–60, 60+ years old, or I prefer not to say?
- Do you have a garden or a balcony, i.e., a garden, a balcony, I have both, or I have neither?
- In your opinion, how important is time outdoors to your health or well-being, i.e., very important, important, neutral, unimportant, or very unimportant?

The median and interquartile range (IQR) (difference between 25th and 75th percentiles) from the 2017 and 2020 survey results were compared.

The chi-square test of independence was carried out to test the null hypothesis for gender and age at significance level α = 0.05 [19].

The environmental data from the PHE solar monitoring network [20] was used to evaluate ambient available erythema dose, H_AE_, for different exposure scenarios presented by the survey results for the lockdown period of April–June 2020. To investigate the variation across different regions of the UK, four sites were considered: Chilton (51°35′, −1°19′), Camborne (50°22′, −5°33′), Belfast (54°60′, −5°83′), and Lerwick (60°14′, −1°19′). Erythema effective irradiance was recorded in the horizontal plane as a 5 min average by RB-501 radiometers (Solar Light Company, Glenside, PA, USA). The H_AE_ was expressed in SED; the CIE Standard Erythema Dose of 100 J/m^2^ [21] (2 SED is approximately the dose to produce perceivable erythema in Fitzpatrick skin type 1 [22]).

Sun exposure during the lockdown could have been impacted by both homeworking and the exceptionally sunny weather in spring 2020. The sunshine hours in spring 2020 were the highest on record across the UK—up to 21 and 35% higher in Scotland and England, respectively, compared to the 1981–2010 average [23]. The increase in sunshine hours cannot provide direct quantitative information on the variation in the short wavelength UV spectral range; sunshine hours are defined by incident direct solar radiation of >120 W m^−2^ [24], and terrestrial solar radiation in the UV range (280–400 nm) is only a fraction of the wavelength range.

To investigate how the spring record high sunshine hours and homeworking could affect UV exposure, the H_AE_ during the lockdown of 2020 was compared to the average values for the same period of 2015–2019 using results of the 2017 survey.

## 3. Results

A total of 657 responders opened the survey and 513 completed it; the response rate was 11.7%, calculated as follows: number of 657 responders per 5584 total staff. Due to organizational restrictions, participants could not be contacted directly and the exact number of responders who read information about the survey in the newsletter and decided not to participate, rather than missing this information altogether, is unknown. 

To investigate how the spring record high sunshine hours and homeworking could affect UV exposure, the H_AE_ during the lockdown of 2020 was compared to the average values for the same period of 2015–2019 using results of the 2017 survey.

The responders were from the following age groups, the PHE staff age-profile data are provided in brackets:

- 3.1%—younger than 25 years old (PHE 7%);
- 44.1%—25–45 years old (PHE 50%);
- 41.0%—46–60 years old (PHE 37%);
- 11.8%—older than 60 years old (PHE 6%).

Of those, 76.1% of responders were female (PHE 68%) and 21.8 were male (PHE 32%).

The low response rate was likely affected by PHE frontline COVID-19 emergency response and potential survey fatigue due to regularly issued monthly staff well-being surveys during the lockdown.

### 3.1. Weekdays

During the lockdown, 74.9% (*n* = 376) of responders spent time outdoors in the morning, significantly higher than 31% (*n* = 273) in 2017. Among these, 61% (*n* = 229) of responders went outdoors before 9:00, 20.5% (*n* = 77) between 9:00–10:00 and 18.4% (*n* = 69) between 10:00–11:00. All times are in British Summer Time (BST), that is, UTC+1. In 2017, 99.4% (*n* = 421) of responders commuted to work before 9:00 with 62.6% (*n* = 264) before 8:00 and, therefore, were outdoors before 8:00. Moreover, 50% of responders who went outdoors after 9:00 did it occasionally. The median of up to 30 min outdoors was the same for 2017 and 2020 (IQR: up to 30 min, 30–60 min).

The percentage contribution of each exposure interval, e.g., 15 min 8:45–9:00, to daily dose H_AE_ was calculated for each location as the monthly average for April–June. April–June monthly variations, as well as the difference between four locations, were insignificant (less than 2%), and the upper limits for each time interval are listed in Table 1.

The prevalent up to 30 min exposure between 8:30–9:00 resulted in the H_AE_ not exceeding 2.5% of the total daily ambient available erythema dose, as shown in Table 1. For responders who were outdoors in the late morning, the H_AE_ was higher, up to 5.7%.

**Figure 1:**
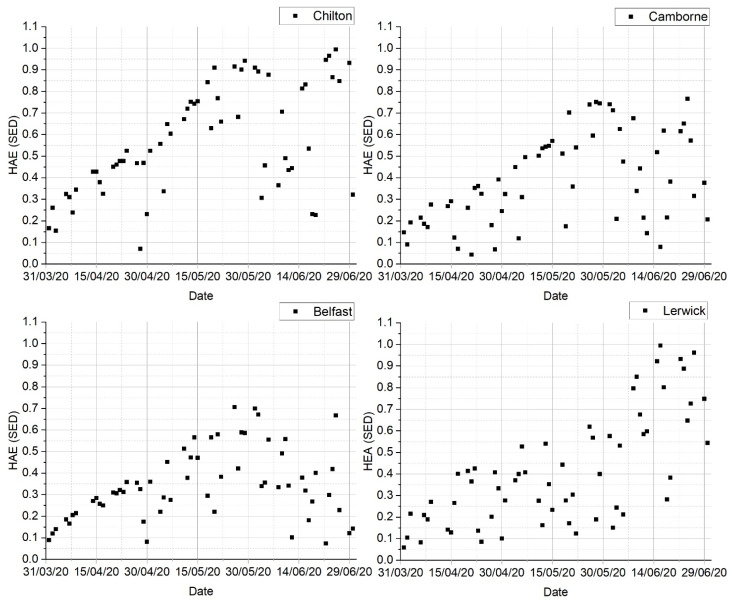
Available erythema effective radiant dose, H_AE_, in 30 min from 8:30–9:00 BST for Chilton, Camborne, Belfast, and Lerwick in April–June 2020.

The 30 min exposures between 8:30–9:00 rarely reached 1 SED in April–June 2020, and they were significantly lower in April, as shown in Figure 1. In June, half of the time the erythema doses were very low at all locations, and the risk of sunburn in the morning was negligible. It should be noted that the H_AE_ presented in Figure 1 relate to the values measured on a horizontal plane; the human body, on an assumption of randomly changing direction, may receive around 30% of this [25], e.g., morning exposure is unlikely to exceed 0.3–0.4 SED, and there was no risk of sunburn at this time of the day.

**Figure 2:**
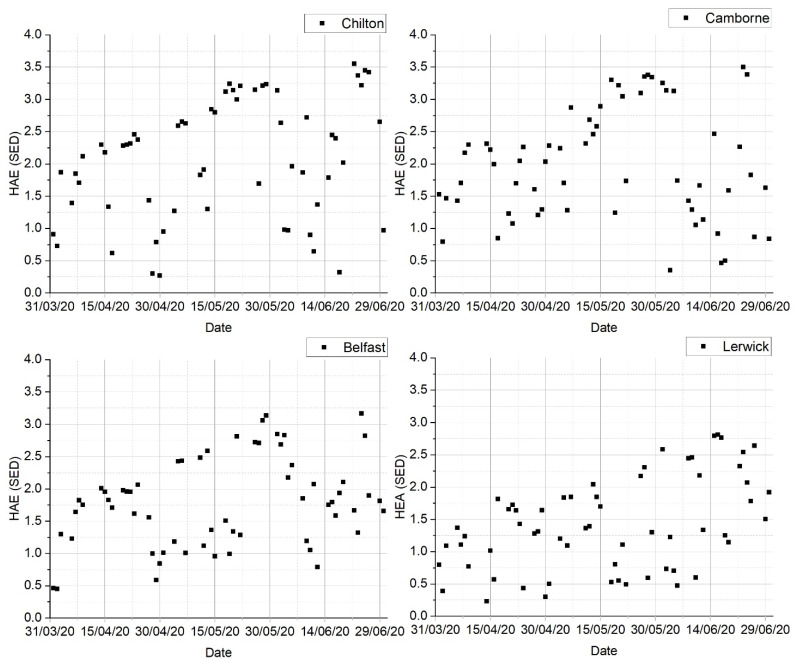
Available erythema effective radiant dose, H_AE_, in 30 min from 12:45–13:15 BST for Chilton, Camborne, Belfast, and Lerwick in April–June 2020.

The lockdown did not significantly change the frequency of time outdoors around midday, which was still governed by work commitments. In 2020, 10.5% (*n* = 52) of responders were always outdoors in midday, compared to 11% (*n* = 98) in 2017; 43.4% (*n* = 217), compared to 52.5% (*n* = 496) in 2017, never or only occasionally did so. The median of 15–30 min outdoors was the same for 2020 and 2017 with IQR (up to 15 min, 30–45 min) and (up to 15 min, 15–30 min), respectively. Additionally, 30 min outdoors around midday could contribute to 8% of the total daily available erythema dose in the lockdown months (Table 1). As shown in Figure 2, the H_AE_ did not exceed 2.5 SED in April across the UK, increasing to ~3.5 SED in later months this year; Lerwick had consistently lower H_AE_ than the rest of the UK. Sun protection was generally needed at midday in April–June 2020 since exposures often exceeded 2 SED, even for relatively short times outdoors.

The biggest difference due to homeworking during lockdown was for the late afternoon (15:00–18:00), and it was gained from not commuting. In 2020, 91.1% (*n* = 444) of the responders spent time outdoors in the late afternoon; 34.2% (*n* = 167) were outdoors ≤30 min and 37.5% (*n* = 183) for 30–60 min (median of 30–60 min and IQR: up 30min, 30–60 min). The estimated 60 min sun exposure could contribute up to 10.6% of the daily H_AE_ if it happens shortly after 15:00 BST, and up to 3.9% after 16:00. The H_AE_ were ~25% lower than midday values presented in Figure 2; they could have reached 5 SED for 60 min outdoors and sun protection was essential. In 2017, only 8% (*n* = 35) of responders left the office before 16:00 and 56.6% (*n* = 251) after 17:00, followed by commuting and thus delaying outdoor time to the evening.

In the evening (after 18:00), 88.7% (*n* = 431) of responders spent time outdoors in the 2020 lockdown, compared to 76.0% (*n* = 665) in 2017. The median was 30–60 min and up to 30 min for 2020 and 2017, respectively (with the same IQR: up 30 min, 30–60 min). Evening exposure only resulted in a negligible H_AE_ of up to 2.4% of the available daily erythema dose.

The time spent outdoors was independent of participants’ age and gender (*p* > 0.05).

To show the difference between spring UV exposure in lockdown and pre-lockdown, the cumulative H_AE_ was calculated for Chilton, Camborne, Belfast, and Lerwick; Camborne was not significantly different from Chilton and, therefore, is not shown here. Based on the middle value of the range of the median of the time spent outdoors (shown in Table 2) and spring 2020 data, the cumulative H_AE_ for the lockdown was estimated and listed in Table 3. As detailed in Table 2, an estimated 105 min outdoors on weekdays during the 2020 lockdown could result in a total H_AE_ between 132.1 SED in Lerwick and 173.2 SED in Chilton, if spent between 8:45–9:00, 15:00–15:45, and 18:00–18:45 BST (no midday exposure). For a small percentage of responders (10.5%) that were outdoors regularly around midday (22.5 min), it could increase by an additional 35%. Delaying time outdoors in the late afternoon to 17:00–17:45 BST or later decreases the cumulative H_AE_ by 32%. 

Based on the results of the 2017 survey listed in Table 2, the cumulative H_AE_ was also estimated using the 2015–2019 average data; values are listed in Table 3 for Chilton, Belfast, and Lerwick. The weekday 30 min outdoors (15 min between 7:45–8:00 and 18:00–18:15 BST, see Table 2) could result in a negligible H_AE_ in all locations. An additional 22.5 min around midday could increase the H_AE_ by 84%.

### 3.2. Weekends and Holidays

The lockdown did not affect time outdoors at weekends; the median of 4–6 h and IQR (2–4 h–8–10 h) were the same for 2020 and 2017 (as shown in Table 2). Based on the middle value of the median range, an estimated contribution of 150 min outdoors around the middle of the day at weekends and on public holidays could result in up to 39.5% of the daily H_AE_ in the period between April–June 2020, reaching the total H_AE_ of 314.4 SED in Chilton, 239.1 SED in Belfast, and 168.0 SED in Lerwick (see Table 3) over the April–June period. The total H_AE_ over the same period based on the 2015–2019 average was 249.7 SED in Chilton, 207.8 SED in Belfast, and 154.8 SED in Lerwick. The weekend erythema doses during the lockdown were higher in all locations by 20.6–13.1% due to the unusually high number of sunny days in 2020, and there was a significant risk of sunburn if sun protection was not used. Since people spent the same amount of time outdoors at weekends in 2020 when it was exceptionally sunny and in the more typical 2017 of the previous survey, it is likely that the sunny weather was not the main reason for a change in behaviour on weekdays, but it was likely due to homeworking in lockdown.

The lockdown travel restriction impacted holidays and travel was limited to essential only; the UK holiday resorts were not permitted to accommodate visitors, and many international flights were suspended. According to the Office of National Statistics, in the lockdown period, there were 96% fewer UK residents’ visits abroad, compared with the same period in 2019 [26]. In spring 2017, 26% of responders went to very high (5–7 days) and extreme (7–14 days) UV Index destinations and 33.4% of responders spent time off in the UK (5–7 days). The majority of responders in 2020 decided not to take or could not take any time off work. Assuming 8 h outdoors between 10:00–18:00 local time, the H_AE_ over one week of holiday in Cyprus or in the UK in the last week of May (UK school half-term week), based on 2015–2019 average data, contributed to an additional 261.2 (Cyprus) and 139.7–96.9 SED (UK), as illustrated in Table 3. In the 2020 lockdown, due to travel restrictions, those additional holiday exposures were unlikely.

## 4. Discussion

Information on the impact of the lockdown on visiting green and natural spaces in England was also collected by the People and Nature Survey (PN) which collects data through an online survey relating to people’s enjoyment, access, understanding of and attitudes towards the natural environment, and its contributions to well-being every month since April 2020 [18]. The PN survey builds on the Monitor of Engagement with the Natural Environment Survey in use from 2009 to 2019 and is representative of adults aged 16+ in key population groups living in England. Although the results of the PN survey cannot be directly compared to this study since green and natural spaces defined in the PN survey excluded private gardens and residential streets, the results from both surveys can be discussed in a broader context. 

Our results showed that 36.2% of responders spent time outdoors every day, compared to 20% in green and natural spaces of the PN study. The difference could be explained by including other locations that are not considered green and natural, e.g., private gardens in the current study: Overall, 86.5% of responders (*n* = 417) had their own or had access to private gardens. Similarly, 46% of PN responders reported that they were spending more time in green and natural spaces than before the COVID-19 pandemic; our results showed that 91.1% of responders were spending more time outdoors in the late afternoon than before the lockdown which may be explained by the prevalence of homeworking in our participants. This was confirmed by the comments in the 2020 study that homeworking allowed respondents to spend more time outdoors than before the lockdown, and as a result, their physical and mental health was better or they felt that time outdoors was directly responsible for coping with the lockdown and increased workload/long hours worked on the COVID-19 emergency response. Moreover, 96.9% of responders in the current survey felt that time outdoors is very important for their health and well-being, and 42% of responders in the PN survey stated that ‘nature and wildlife are more important than ever to their well-being’. Some responders shared behavioural changes they faced, such as spending more time outdoors due to gyms being closed, spending coffee breaks outdoors, incorporating walking teleconferences when possible, or/and working in the garden. In the PN survey, 33% of responders said that they had more visits to local green and natural spaces than before the pandemic. In our study, 9.9% of responders (*n* = 51) did not go outdoors on weekdays due to the workload of the COVID-19 emergency response, shielding themselves or family members. The workload was the most common reason for spending less or no time outdoors on weekdays. Some people avoided leaving the house because others did not observe the social distancing rules. The PN survey showed that 23% of responders had not spent any time in green and natural spaces in the previous two weeks due to concerns about contracting or spreading coronavirus (43%) or breaking coronavirus restrictions (26%).

The strength of the current study is that the survey took place during a period of significant restrictions; thus, the data gathered are contemporaneous. These data were compared with the results of the survey carried out in 2017 and do not rely on recalling behaviours in the lockdown-free period. Additionally, data were analysed on substantially the same cohort; responders were pulled from the same sample to measure the effect of the lockdown and homeworking on time spent outdoors. 

The main limitation of the study is that respondents were neither randomly selected from the UK adult population nor weighted to UK demographics. Therefore, the data cannot be directly interpreted as statistically representative of the UK adult population.

Due to the comparable but low response rate in the 2017 and 2020 surveys (11.7% in 2020 and 16% in 2017), an element of bias may also be inherent in these surveys: it is likely that participants with an interest in outdoor activities were more likely to respond. A high percentage of homeworking PHE staff also may not be representative of the wider UK population.

In this observational study, accurate quantitative analysis of vitamin D synthesis was not feasible since recorded information on time outdoors did not differentiate where and how this time was spent, e.g., in the open unshaded areas or shaded by vegetation, such as parks, woodlands, or private gardens; percentage of bare skin area or use of sun protection could only be hypothesised but was not recorded. Similarly, the relatively short timescale of this study, 3 months of lockdown only, and the exceptional circumstances of national lockdown are insufficient for quantitative evaluation of skin cancer risks, in particular, assessment of relative contributions of potentially higher chronic daily exposures due to exceptionally sunny spring 2020 counterbalancing the absence of intermittent very high exposures from overseas holidays. Such future analysis is very important since it is expected that homeworking will continue beyond lockdown restrictions. 

It is expected that homeworking will continue beyond the COVID-19 pandemic. It offers more flexibility and substantially reduces the burden of long commutes; more people could be encouraged to spend more time outdoors and change to a more active lifestyle. Many responders already recognised the importance of spending time outdoors for mental health and well-being. The analysis showed that homeworking could increase weekday UV exposure by up to 50% modelled on the 2015–2019 average more typical weather conditions. Longer sun exposures in the middle of the day and early afternoon in spring and summer as well as on holiday would require sun protection to avoid overexposure.

The pattern of behaviour may be different after lockdown restrictions are eased and holiday travel resumes; the impact of health risks and benefits will need further consideration which should include demographic inputs, temperatures, accessibility to outdoor spaces, etc., in order to draw conclusions with sufficient confidence. 

## 5. Conclusions

The impact of the UK lockdown due to the COVID-19 pandemic in April–June 2020 on UV exposure of office workers was assessed using an online-based survey on time spent outdoors and environmental data for four locations in the UK. Three aspects of this lockdown were considered: homeworking resulting in increased flexibility and substantially reduced commuting, the exceptionally sunny spring in 2020, and travel restrictions that impacted overseas holidays from April–June 2020.

Without the need for commuting and with the flexibility of homeworking, weekday sun exposure was significantly higher in 2020 than in 2017. This increase originates from an additional 45 min outdoors in the late afternoon and high UV levels due to extremely sunny weather in spring 2020. The lockdown did not impact the frequency of time spent outdoors around midday which was still governed by work commitments. Overall, 9.9% of responders spent no time outdoors on weekdays due to the workload of the COVID-19 emergency response or shielding. At the weekends, no difference in time spent outdoors between 2020 and 2017 was observed. In 2020, responders felt that time outdoors is very important for their health and well-being. Some commented that homeworking allowed them to spend more time outdoors than before the lockdown, and as a result, their physical and mental health was better or they felt that time outdoors was directly responsible for coping with the lockdown and increased workload/long hours worked on the COVID-19 emergency response. Results of this study could also provide important insight to research on exposure to sunlight for other health markers, such as, for example, melanopic exposure for circadian entrainment.

It is expected that homeworking will continue beyond the COVID-19 pandemic. Homeworking offers more flexibility and substantially reduces the burden of long commutes and more people could be encouraged to spend more time outdoors and engage in a more active lifestyle that may positively impact productivity, and mental health and well-being. However, it also emphasises the importance of sun protection if outdoor activities increase.

## Figures and Tables

**Table 1:** Contribution of exposure at the different times of day to the daily ambient available H_AE_ for different exposure scenarios in April–June.

Duration	Timing	% of the Daily H_AE_
Morning		
15–30 min	08:30 to 09:00	≤2.5%
15–30 min	9:30 to 10:00	≤4.3%
15–30 min	10:30 to 11:00	≤5.7%
Midday		
15–30 min	12:15 to 12:45	≤8.6%
150 min	11:45–14:15	up to 41.4%
Afternoon		
30–60 min	15:00 to 16:00	≤12.2%
Evening		
30–60 min	18:00 to 19:00	≤2.7%

**Table 2:** The median time spent outdoors and its timing according to 2020 and 2017 surveys. Time is given in BST; except holidays in Cyprus.

Weekdays	2020 Survey (*n* = 513)	2017 Survey (*n* = 894)
Morning	15 min 8:45–9:00	15 min 7:45–8:00
Midday	22.5 min	
Afternoon or later afternoon	45 min: 15:00–15:45 or 45 min: 17:00–17:45	none
Evening	45 min: 18:00–18:45	15 min: 18:00–18:15
Weekend	300 min: 11:45–14:15	
Holidays in the UK	none	8 h: 10:00–18:00
Holidays in Cyprus	none	8 h: 10:00–18:00 local time

**Table 3:** H_AE_ (in SED units) in unshaded outdoors based on April–June 2020 and the 2015–2019 average data for different exposure scenarios in Chilton, Belfast, and Lerwick. Note that H_AE_ relates to the values measured on a horizontal plane and the human body may receive around 30% of these values on an assumption of randomly changing orientations. * Later afternoon exposure; ** total available erythema dose including a holiday in Cyprus.

	H_AE_ in Chilton, SED		H_AE_ in Belfast, SED		H_AE_ in Lerwick, SED	
Weekdays	2020	Average 2015–2019	2020	Average 2015–2019	2020	Average 2015–2019
Mornings	19.2	6.6	11.8	3.9	13.5	5.6
Middays	94.5	75.9	81.9	62.2	65.1	52.0
Afternoons	131.5	---	123.6	---	96.4	---
Later afternoon	49.5 *	---	51.0 *	---	42.9 *	---
Evenings	22.5	7.4	24.6	8.4	22.2	6.2
Total weekdays	267.7/185.7 *	89.9	241.9/169.3 *	74.5	197.2/143.7 *	63.8
Weekends	314.4	249.7	239.1	207.8	168.0	154.8
Holidays in the UK	---	139.7	---	119.2	---	96.9
Holidays in Cyprus **	---	261.2 **	---	261.2 **	---	261.2 **
Total including holiday	582.1/500.1 *	479.3/600.8 **	481.0/408.4 **	401.5/543.5 **	365.2/311.7 **	315.5/479.8 **

The total weekday H_AE_ was higher during the 2020 lockdown by up to 69.0 and 56.0% for responders who spent time outdoors around midday and in the afternoon between 15:00–15:45 and 17:00–17:45, respectively.

## Data Availability

PHE solar network data are available under the Open Government License.
